# Dataset on bacterial diversity using 16S metagenome analysis in fermented rice beer from two breweries and grape wine of Mizoram, Northeast India

**DOI:** 10.1016/j.dib.2024.110932

**Published:** 2024-09-11

**Authors:** Benjamin Lalbiakmawia, Sowmya Pulapet, Sowmiya Kathir, R. Lalengkimi, Kesavan Markkandan, Michael V． L. Chhandama, Senthil Kumar Nachimuthu, John Zothanzama

**Affiliations:** a Department of Biotechnology, Mizoram University, Tanhril, Aizawl, Mizoram 796004, India; b ONEOMICS PRIVATE LIMITED, Bharathidasan University Technology Park, Khajamalai, Campus, Tiruchirappalli, Tamil Nadu 620023, India

**Keywords:** Metagenome, Bacterial diversity, Next-generation sequencing, 16S rRNA, Traditional rice beer

## Abstract

The microbial diversity of fermented rice beer and grape wine in Mizoram was explored using 16S metagenome analysis. The collected samples were marked as C1 and B1 for fermented rice beer and D1 for grape wine. Next-generation sequencing of the 16S rRNA (V3–V4 region) was performed using the Illumina NovoSeq 6000 platform. Operational taxonomic units (OTUs) were identified with QIIME2, and statistical analyses were performed using R packages. The metagenome of the three samples comprised 464,826 raw reads that represented 116,206,500 base pairs and were clustered into 336 OTUs. The phylum Firmicutes was predominant in C1 (55 %), B1 (53 %) and D1 (52 %), respectively and biosysnthesis, pyruvate fermentation to be abundant functions. By applying 16S metagenome analysis, this data provide insights in to the complex community of bacteria involved in the fermentation process and their potential roles and interactions.

Specification Table

**Table:** 

Subject	Agricultural Sciences, Food Science: Food Microbiology
Specific subject area	Identification and characterisation of microorganisms present in the traditional drinks of native rice beer (Zufang) and wine of Mizoram, Northeast India
Type of data	Table, Graph and Figure Raw, Analyzed, Filtered
Data collection	Samples were sourced from two local breweries. DNA was extracted using the modified method followed in ONEOMICS PRIVATE LIMITED, TIRUCHIRAPPALLI, TAMIL NADU, INDIA. Next-generation sequencing of the 16S rRNA (V3–V4 region) was performed on the Illumina NovoSeq 6000 platform The libraries were quantified via Qubit 4.0 and qPCR and sequenced using NovoSeq 6000 with 250 bp paired-end chemistry. The raw reads for the samples were 110,632, 230,398 and 123,796 for B1, C1 and D1, respectively. The library was constructed using the Quick-16S NGS Library Prep Kit, as described in the manufacturer instructions. The 16S rRNA reads generated were processed using the Quantitative Insights into Microbial Ecology v2020.2 (https://qiime2.org/) pipeline.
Data source location	The samples (B1, C1 and D1) were collected from Aizawl, Mizoram, India (23°43′38″N 92°43′04″E). DNA isolation, sequencing and data analysis were performed in Mizoram University, Mizoram and ONEOMICS PRIVATE LIMITED, TAMIL NADU, INDIA.
Data accessibility	Repository name: NCBI Bioproject, SRA Data identification number: PRJNA1008667, SRX21467463, SRX21467462, SRX21467461 Direct URL to data: https://www.ncbi.nlm.nih.gov/sra/?term=PRJNA1008667 https://www.ncbi.nlm.nih.gov/bioproject/?term=PRJNA1008667 Data can be accessed from the NCBI site by entering the data identification number in the search bar.

## 1 Value of the Data

• The data provides information on the microbial diversity of traditional rice beer and grape wine from the state of Mizoram, Northeast India, and offers basic insights into the vast diversity of fermented drinks in the region. The data are likely to be beneficial for performing comparisons with the microbial diversity of other rice beer samples.
• Significant microbial diversity was observed in rice beer compared with grape wine.
• The data offers the possibility of discovering novel bacteria that have previously not been reported in fermented rice beer.

## 2 Background

For several years, fermented drinks have been an important part of the communities in Northeast India. The methodology of starter cake preparation and the incubation time for batch culture of rice beer differ significantly among the tribes. One such traditional rice beverage of the native tribal communities of Mizoram is Zufang. The rich microbial communities contribute significantly to product quality, taste and flavour and may benefit the consumer's health. Hence, monitoring these communities is crucial. Conventional microbiological tests used in breweries include microscopy and cultivation-based approaches. However, these methods are quantitatively imprecise and capture only a fraction of the microbiome diversity [1]. By applying 16S metagenomic analysis, this study aims to shed light on the intricate microbial communities that contribute to the fermentation process of rice beer. For 16S metagenomic analysis, the V3–V4 region which is highly variable and is known to reveal microbial diverse populations, is used [3]. This region of the 16S rRNA gene is advantageous for sequencing on platforms such as Illumina owing to its effective balance of taxonomic resolution and diversity analysis, suitability for high-throughput sequencing methods, cost-efficiency, comparative advantages over other hypervariable regions, and broad applicability across different ecological studies [6].

## 3 Data Description

A total of 464,826 reads were generated from the three samples: C1 and B1 for fermented rice beer, and D1 for grape wine. The high-quality reads were clustered to form 336 operational taxonomic units (OTUs), as shown in Table 1.

**Table 1:** Sequencing statistics of rice beer (B1, C1) and grape wine (D1).

Sample	Raw reads	Sequences (bp)	GC (%)	Q30 score
B1	110,632	27,658,000	51	33.2
C1	230,398	57,599,500	52	31.4
D1	123,796	30,949,000	53	31.3

The data were deposited at the repository.

Raw sequences of the samples were uploaded to NCBI BioProject SRA with the data identification number PRJNA1008667, as described below:

‐ 16S Metagenome Analysis in Fermented Grape Wine of Mizoram, Northeast India, Raw sequence reads 1 ILLUMINA (Illumina NovaSeq 6000) run: 61,898 spots, 30.9 M bases, 17.2Mb downloads Accession: SRX21467463
‐ 16S Metagenome Analysis in Fermented Rice Beer 2 of Mizoram, Northeast India, Raw sequence reads 1 ILLUMINA (Illumina NovaSeq 6000) run: 66,748 spots, 33.4 M bases, 19Mb downloads Accession: SRX21467462
‐ 16S Metagenome Analysis in Fermented Rice Beer 1 of Mizoram, Northeast India, Raw sequence reads 1 ILLUMINA (Illumina NovaSeq 6000) run: 55,316 spots, 27.7 M bases, 14.8Mb downloads Accession: SRX21467461.

**Fig. 1:**
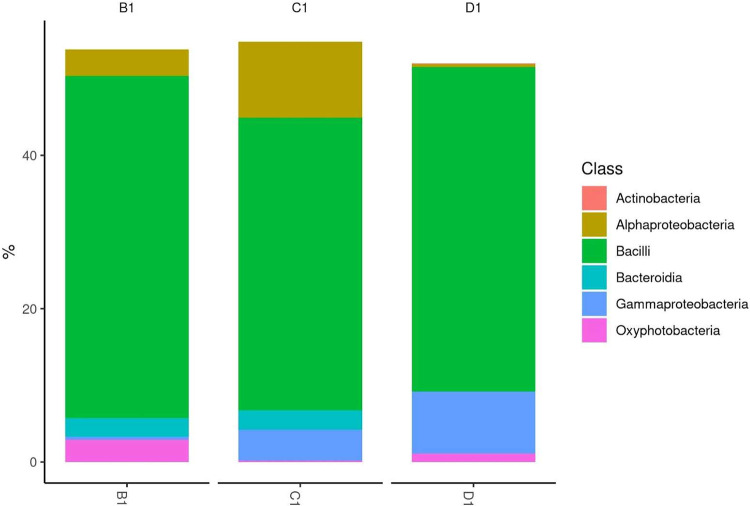
Relative abundance of dominant bacterial phyla in B1, C1 and D1.

The detected OTUs were divided into 5 phyla, 6 classes, 14 orders, 18 families and 21 genera. Of the phyla identified, Firmicutes were prominent in all three samples. Other phyla included Bacteroidetes, Actinobacteria and others, with <0.50 % of the reads. A total of 14 orders were recorded in all three samples, and only Lactobacillales exhibited a relative abundance of >99 % (Fig. 1).

**Fig. 2:**
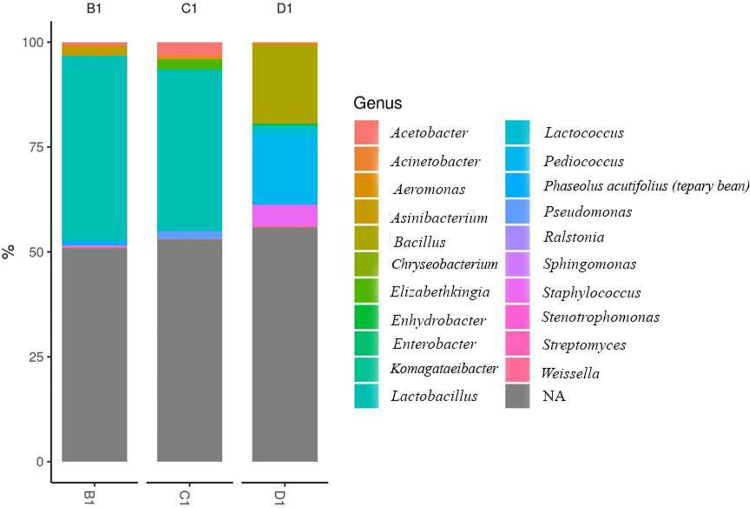
Relative abundance of the dominant bacterial genera in B1, C1 and D1.

The difference in the abundance of genera between the samples is depicted with the help of an abundance bar plot (Fig 2). At the genus level, Lactobacillus was abundant in the rice beer samples (B1 - 44 %, C1 – 38 %). The bacteria like Bacillus subtilis, known to produce enzymes that hydrolyse complex carbohydrates into simple sugars, thus facilitating fermentation and enhancing the nutrient availability for other microorganisms were identified.

**Fig. 3:**
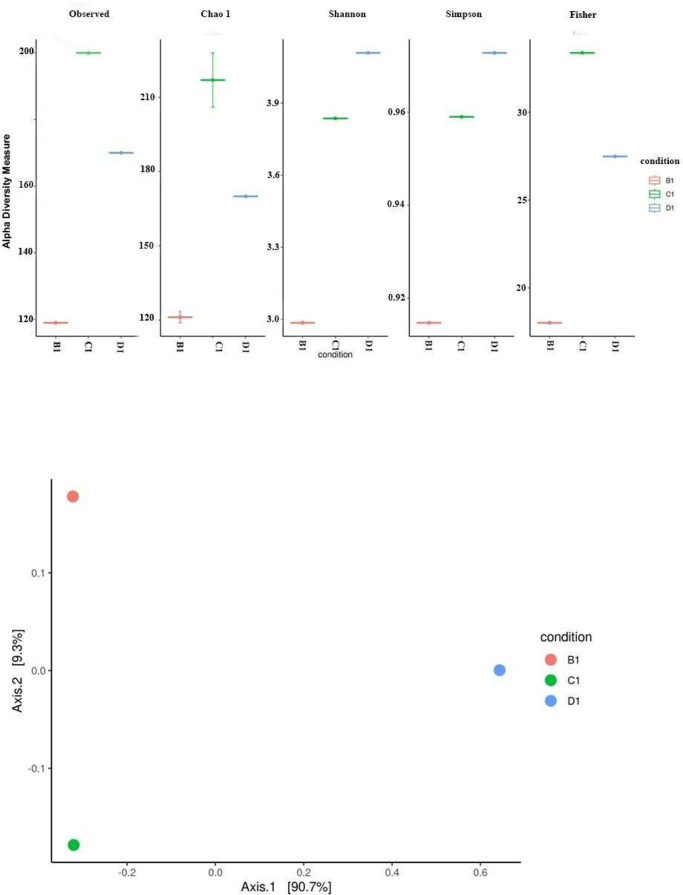
Diversity analysis of the samples B1, C1 and D1. (a) Alpha diversity (b) Beta diversity.

The diversity comparison for the three samples is given in Fig. 3. The Chao1, Shannon and Fishe r indices were used to represent the variation in diversity among the samples.

**Fig. 4:**
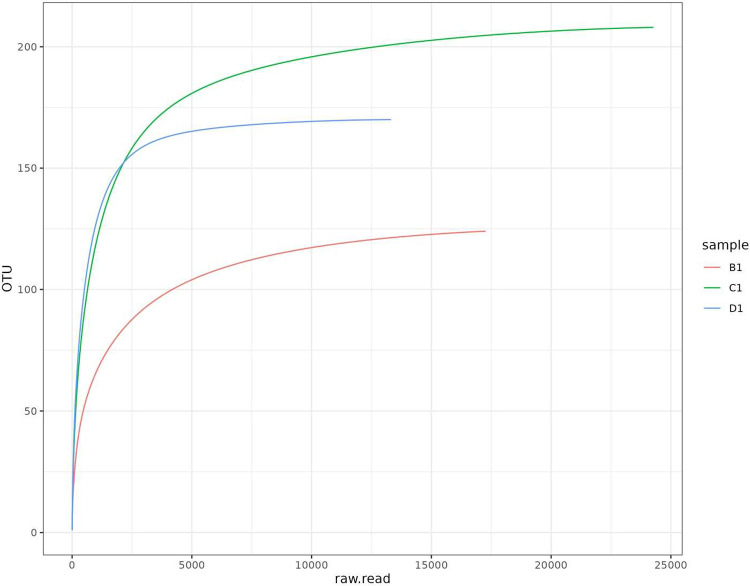
Rarefaction curves of samples B1, C1 and D1.

The rarefaction curve is generated showing the correlation between the number of reads and the number of OTUs identified (Fig. 4)

**Fig. 5:**
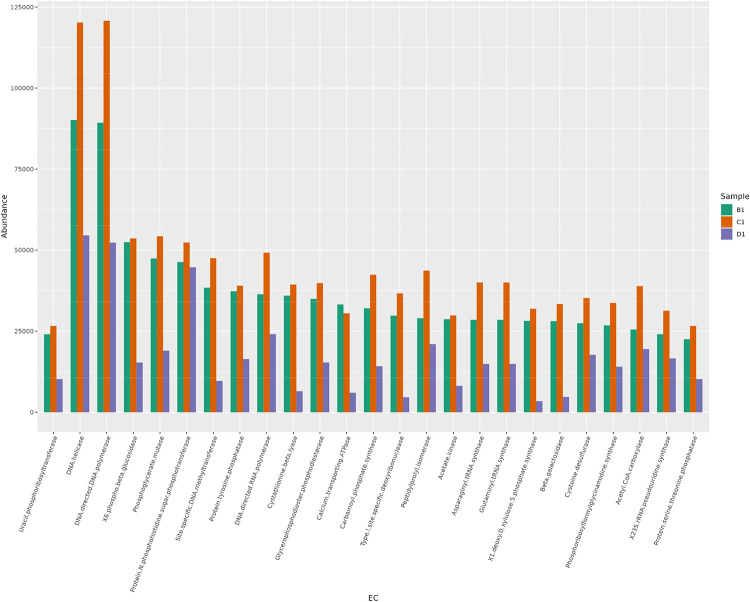
Bar plot of Picrust2-predicted EC annotations for samples B1, C1 and D1.

To understand the predicted functional modules associated with the samples, PICRUSt2 was performed using the 16S rRNA datasets. The EC annotation was obtained for the samples (Fig. 5), to identify enzymes present in the samples.

**Fig. 6:**
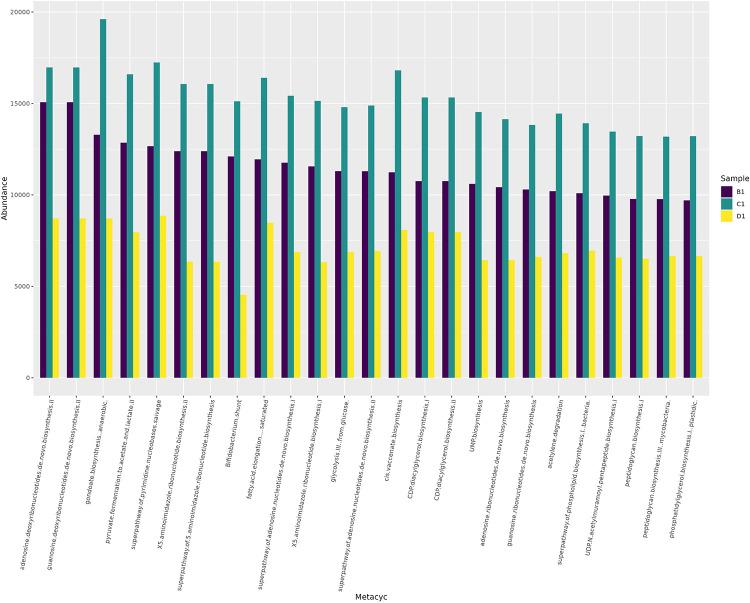
Bar plot of Picrust2-predicted MetaCyc pathways for samples B1, C1 and D1.

The predicted MetaCyc pathways (Fig. 6) included nucleotide biosynthesis, pyruvate fermentation and energy production pathways.

## 4 Experimental Design, Materials and Methods

### 4.1 Research design

The study aims to assess the intricate microbial communities that contribute to the fermentation process of rice beer and predict microbial metabolites function. It is an exploratory research using quantitative approach focusing on the microbial diversity and metagenomics analysis.

The study follows a purposive sampling design to assess the microbial diversity in the tribal fermented beverage ‘Zufang’ in comparison with the commercial wine produce in the area. DNA was extracted from these samples where V3-V4 region of 16S rRNA region was targeted as it is highly variable and is known to reveal microbial diverse populations [3]. Library was prepared using Quik 16s Library Prep kit and sequence using Illumina NovoSeq 6000 platform for cost effectiveness, efficiency and accessibility.

For bioinformatics analysis 16S rRNA reads were processed using QIIME2 pipeline and sequences were classified against SILVA v132 database to generate alpha and beta diversity of the micro biome and statistical analysis was used to compare microbial diversity between the samples. These bioinformatics tools are used in tandem to processed sequences, taxonomic classification, diversity analysis, functional prediction and visual data generation. The use of these tools ensures a robust and cost effective analysis that is easily replicable, providing further insights on micro biome of fermented drinks.

### 4.2 Sampling

#### 4.2.1 Preparation of rice beer (Zufang)

Mizo sticky rice (buhban) grown in the state and belonging to the kawng lawng (*Oryza sativa****)*** variety is usually used to prepare Zufang. A starter yeast (*Saccharomyces cerevisiae)* culture (chawl), which is a commercial culture imported from Myanmar, is sprinkled over the cooked sticky rice and fermented in a special dedicated vessel called ‘Zufang bel’. Zufang is a sweet beverage with a mild alcoholic content and undissolved rice and is consumed traditionally by the Mizo tribal people. Sampling was done from two different locations (B1 and C1 samples) of Aizawl where Zufang is prepared regularly. For the preparation of Zufang, 2–5 kg of rice is cooked and cooled in a sterile room. The starter yeast culture is mixed with the cooled rice and allowed for fermentation in a suitable covered container. The container is kept in sunlight for 3–4 days in summer and for 5–6 days in winter for fermentation. Subsequently, the liquid is either directly consumed or distilled. The fermentation temperature is not specified for locally produced Zufang. In the current study, the samples were collected in the month of August during which the average ambient temperature is 30°C. A noteworthy point is that instead of the sticky rice, regular normal rice of the Indian variety was used by the local brewer for sample C1. Sterilized clean bottles were immersed in the brewing pot and filled with the samples after the fermentation was done for 3 days.

#### 4.2.2 Isabella wine-making process

The grape variety used for Isabella wine is *Vitis labrusca* grown locally in the state. It is well known that Mizoram soils are acidic in nature, with average pH ranging from 5.5 to 6.0 [5]. The harvested grapes are crushed using a grape destemmer–crusher. Sugar is then added to the grape juice thus extracted to obtain a high Brix value as the original Brix value of the harvested grapes (Isabella) is only approximately 14. The grape juice is then fermented for about 1 week in a stainless-steel fermenter with the addition of yeast (*Saccharomyces cerevisiae*). During the fermentation, timed punch downs occur for several days. After the wine has been fermented and punched for the appropriate period, the skins and seeds are separated by filtration. The filtered wine is then bottled for marketing. The sample (D1) was purchased from the local market.

### 4.3 DNA extraction

Genomic DNA was isolated from the rice beer and grape wine samples using the modified method followed in ONEOMICS PRIVATE LIMITED, TIRUCHIRAPPALLI, TAMILNADU. In this procedure, 200 µL of the samples was mixed with an equal volume of high-salt lysis buffer. The tube was vortexed vigorously and placed at 55 °C for 10 min. To this mixture, an equal volume of phenol:chloroform:isoamyl alcohol was added, followed by centrifuged at maximum speed. The supernatant was pipetted out, and two volumes of 100 % ethanol was added and subjected to DNA precipitation at −20 °C. Finally, the tube was centrifuged at maximum speed, and the pellet was washed with ethanol. Following isolation, the DNA concentrations were verified using a Qubit 4.0 Fluorometer (Thermo Fisher Scientific, USA) and the DNA quality was determined using Nanodrop and 0.8 % (w/v) agarose gel electrophoresis.

### 4.4 Library construction and illumina sequencing

The library was prepared using the Quick-16S NGS Library Prep Kit, as described in the manufacturer instructions. Briefly, the variable region of the 16S rRNA gene (V3–V4) was amplified using specific primers (341F -CCTAYGGGRBGCASCAG and 806R - GGACTACNNGGGTATCTAAT) along with the barcode using PCR. All PCR reactions were performed with the Phusion® High-Fidelity PCR Master Mix (New England Biolabs). The PCR products was mixed in equidensity ratios and purified with the Qiagen Gel Extraction Kit (Qiagen, Germany), followed by a run on 2 % (w/v) agarose gel. The amplified PCR product of 460 bp was selected for further experiments. The libraries were quantified via Qubit 4.0 and qPCR and sequenced using NovoSeq 6000 with 250 bp paired-end chemistry.

### 4.5 Bioinformatic analysis

The 16S rRNA reads generated were processed using the Quantitative Insights into Microbial Ecology v2020.2 pipeline [2]. The reads were first demultiplexed, and the pairs were joined. The sequencing errors and noisy sequences were removed using the Deblur algorithm. The sequences were clustered based on similarity and aligned against a trained Naïve Bayes classifier (SILVA v132) [4] for taxonomy classification using the q2-feature-classifier plugin. The alpha and beta diversity metrics were calculated in QIIME2 to determine the composition of the bacterial community. The alpha diversity was measured using the observed number of OTUs, Chao1, Shannon, Simpson and Fisher statistical indices. The beta diversity was measured using the Bray–Curtis index, which assesses the compositional dissimilarity. In addition, principal component analysis was performed, and the phylogenetic tree was constructed to visualize the samples. Further visualisation was done using the qiime2R 0.99.6 and phyloseq v1.44.0 R packages. The microbial functional potential was predicted using the Phylogenetic Investigation of Communities by Reconstruction of Unobserved States (PICRUSt2) v2.5.2 to predict the microbial metabolic functions.

## Limitations

Not applicable.

## Ethics Statement

The current work does not involve human subjects, animal experiments, or any data collected from social media platforms.

## CRediT Author Statement

**Benjamin Lalbiakmawia:** Sampling, Conceptualization, Methodology, Original draft preparation. **Sowmya Pulapet:** Data analysis, Data curation, Original draft preparation. **Sowmiya Kathir:** Data analysis, Data curation, Validation. **R. Lalengkimi:** Supervision. Kesavan Markkandan: Review and Editing. **Michael V L Chhandama:** Review and Editing. **Nachimuthu Senthil Kumar:** Review and Editing, Supervision. **John Zothanzama:** Review and Editing, Conceptualization, Methodology.

## Data Availability

16S Metagenome Analysis in Fermented Grape Wine of Mizoram, Northeast India Raw sequence reads (Original data) (NCBI - SRA). (NCBI). SRA (Original Data) SRX21467463(NCBI), SRX21467462(NCBI), SRX21467461(NCBI).
